# Managing Single-Stranded DNA during Replication Stress in Fission Yeast

**DOI:** 10.3390/biom5032123

**Published:** 2015-09-18

**Authors:** Sarah A. Sabatinos, Susan L. Forsburg

**Affiliations:** 1 Department of Chemistry and Biology, Ryerson University, 350 Victoria Street Toronto, ON M5B 2K3, Canada; E-Mail: ssabatinos@ryerson.ca; 2 Program in Molecular and Computational Biology, University of Southern California, 1050 Childs Way, Los Angeles, CA 90089, USA

**Keywords:** DNA replication, single-stranded DNA, replication stress, genome stability, RPA, *Schizosaccharomyces pombe*, checkpoint, MCM, helicase

## Abstract

Replication fork stalling generates a variety of responses, most of which cause an increase in single-stranded DNA. ssDNA is a primary signal of replication distress that activates cellular checkpoints. It is also a potential source of genome instability and a substrate for mutation and recombination. Therefore, managing ssDNA levels is crucial to chromosome integrity. Limited ssDNA accumulation occurs in wild-type cells under stress. In contrast, cells lacking the replication checkpoint cannot arrest forks properly and accumulate large amounts of ssDNA. This likely occurs when the replication fork polymerase and helicase units are uncoupled. Some cells with mutations in the replication helicase (*mcm-ts*) mimic checkpoint-deficient cells, and accumulate extensive areas of ssDNA to trigger the G2-checkpoint. Another category of helicase mutant (*mcm4-degron*) causes fork stalling in early S-phase due to immediate loss of helicase function. Intriguingly, cells realize that ssDNA is present, but fail to detect that they accumulate ssDNA, and continue to divide. Thus, the cellular response to replication stalling depends on checkpoint activity and the time that replication stress occurs in S-phase. In this review we describe the signs, signals, and symptoms of replication arrest from an ssDNA perspective. We explore the possible mechanisms for these effects. We also advise the need for caution when detecting and interpreting data related to the accumulation of ssDNA.

## 1. Introduction

**Figure 1:**
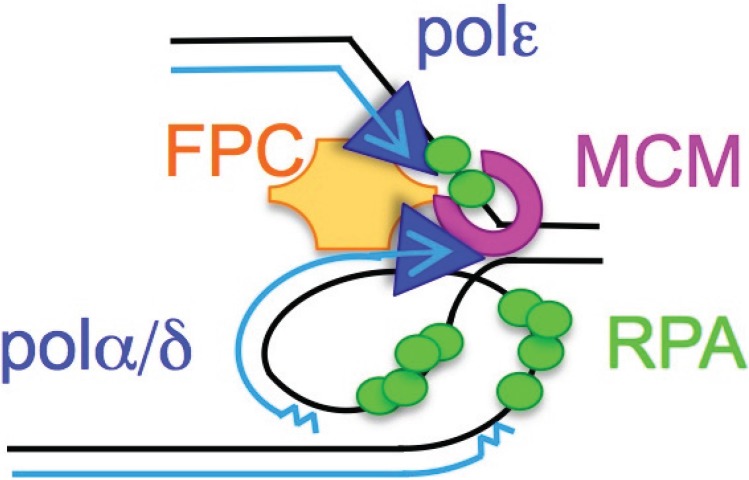
Cartoon of the replication fork showing the replicative polymerases (Polε, Polα, Polδ), the MCM helicase, single-stranded DNA binding protein RPA, and the fork protection complex (FPC) that helps couple components together. The FPC includes molecules TIMELESS (ScTof1, SpSwi1), TIPIN (ScCsm3, SpSwi3), CLASPIN (yeast Mrc1), and AND1 (ScMcf4, SpMcl1). The assignment of leading (Polε) and lagging (Polα/δ) strand polymerases remains controversial, since (Polδ) has roles copying both leading and lagging strands [4]. Numerous additional factors at the replication fork are omitted for simplicity; see [5].

DNA replication stress is a significant contributor to genome instability in cancer and other diseases [1,2,3]. A general definition of replication stress is a condition that impairs the processivity of the normal replisome (Figure 1). Many forms of replication stress are documented, most of which trigger the Intra-S phase checkpoint to stabilize and repair stalled replication forks. Without stabilizing and repairing the causes and effects of replication stress, the genome becomes vulnerable to damage, mutation, or rearrangement. These three factors, singly or in combination with each other, describe the concept of genome instability. Genome instability carries a potential for chromosome mis-segregation and further damage to the genome and the organism.

Many intrinsic conditions contribute to replication stress, such as late replicating regions of the genome, repetitive sequences, and collisions between the DNA replication and gene transcription mechanisms (reviewed in [6]). Stress-associated domains within the genome may vary with cell type and cell program, and can be epigenetically regulated. Frequently, replication stress regions define chromosome fragile sites (CFS) that are particularly prone to breakage and associated with accumulation of ssDNA [7]. Indeed, these may contribute to formation of ultrafine anaphase bridges (UFBs) during mitosis. UFBs are proposed to be threads of ssDNA linked to under-replicated DNA at fragile sites (e.g., [8,9]). CFS regions are also sensitive to external factors, such as drugs that inhibit DNA replication, disruptions in ribonucleotide metabolism, and oncogene activation [1,6,10]. Not surprisingly, mutations affecting proteins that replicate and repair DNA are also linked to cancer, neurological disorders, aging, and developmental defects (reviewed in [6]).

Whether intrinsic or extrinsic, these insults generate replication fork stress, and can lead to genetic instability. How do cells survive replication fork stress? The classic cell cycle model tells us that replication-associated damage activates a checkpoint-signaling pathway to arrest the cell cycle and promote repair. The checkpoint response should facilitate fork restart, recovery, and re-entry into the cell cycle, or promote apoptosis if the damage is irreparable (reviewed in [11,12,13]). The formation of single-stranded DNA (ssDNA) is a crucial effector in the response to replication stress.

## 2. ssDNA Is a Hallmark of Stress

Studies in multiple model systems show that ssDNA accumulation begins during replication fork arrest, when the replicative helicase and DNA polymerases become uncoupled. This leads to increased unwinding of the DNA [14,15,16,17,18,19,20,21,22,23]. However, cells without a functional replication checkpoint response cannot limit this signal. Whereas checkpoint-competent cells limit ssDNA accumulation to a few hundred base pairs, checkpoint mutants form additional ssDNA that may span 1 kb of genome between the helicase and polymerases [23,24,25,26,27].

The ssDNA accumulation can occur on both strands, for example, if there is unregulated unwinding ahead of a replication fork exposing both leading and lagging strands, or uncoupling of the polymerase(s) from the helicase. ssDNA may also occur if there is resection during homologous recombination. The accumulation of ssDNA is associated with increased rates of clustered point mutations in yeast and cancer cell lines [28]. Such clustered mutations suggest that exposed ssDNA is at risk for transient hypermutability that may be clinically relevant.

Disease initiation and progression may also be attributed to additional sources of ssDNA. For example, resection from broken DNA ends, replication fork regression, D-loops from recombination strand invasions, and collision between replication and transcription complexes that generates R-loops (reviewed in [6,11,29]). A failure to manage ssDNA can have serious consequences. Replication stress-associated double strand breaks are preceded by increased ssDNA [30]. Increased expression of DNA cytosine deaminases such as APOBEC, which specifically target ssDNA, is observed in some cancers [31]. Even low levels can be dangerous. Cytidine deaminase converts cytidine to uracil, resulting in C > T and C > G mutations. (e.g., [32]). Clusters of APOBEC mutational signatures described in cancer cell lines led to the development of a new descriptor: “kataegis” (Greek for “thunder”) [33,34].

Furthermore, there is evidence that cells with low levels of ssDNA can evade checkpoints, leading to abnormal mitosis, lagging chromosomes, and anaphase bridges [8,29]. These mitotic abnormalities can cause aneuploidy and overall decreased cell survival. Even so, the cells that manage to survive DNA replication stress are at risk for hyper-mutation and genome rearrangements (e.g., [35]) Therefore, managing ssDNA dynamics during genome stress is crucial to cell survival.

An under-investigated question is whether ssDNA is packaged as chromatin. Typically, regions of ssDNA, such as ultrafine anaphase bridges, can be recognized by protein binding, including the ssDNA binding protein RPA (see below), but not by histone markers (e.g., [35]). However, over 30 years ago, Alberts and colleagues suggested a model of histone octamer-ssDNA interactions that maximize charged contacts between ssDNA and histones and hold the ssDNA in place [36]. In their model, a single strand of ssDNA wrapped in a dsDNA-like conformation around a histone does not maximize charged contact zone contacts. Therefore, the interaction between ssDNA and the histone octamer is loose. Instead, they proposed two potential preferred conformations for ssDNA in a nucleosome. In the first, the ssDNA wraps almost halfway around the octamer before looping out and winding back onto the histone in a reverse direction. The second model suggests that two separate ssDNA molecules could enter mid-way along a nucleosome and wind to exit in opposite orientations, thus sharing a single nucleosome (Palter *et al.*, 1979 [37]). The first case suggests that a longer ssDNA molecule is wrapped in a nucleosome. The second prediction might describe a situation at a DNA double strand break, particularly if a nearby octamer were co-opted to maintain ssDNA stability by balancing its charged surfaces.

These predictions have not been explored but are potentially important to our understanding of how nucleosomes repopulate replicated DNA. While little is known about ssDNA structures in chromatin, crystal structures argue that dsDNA-histone interactions are dependent on both the DNA-strand that contacts the histone (e.g., Watson or Crick strand), and the histone-protein sequence at the contact point [37]. Therefore, ssDNA *versus* dsDNA may have different opportunities to associate with histones. Further, charge changes to histone proteins may alter the octameric structure and affect DNA binding [37]. Clearly, any histones associated with ssDNA cannot interfere with normal ssDNA metabolism, either because they are restricted to certain regions or structures, or because the nature of the interaction leaves the ssDNA accessible.

## 3. RPA Is the ssDNA Sensor

The central ssDNA binding protein of eukaryotes is the trimeric replication protein A complex (RPA). RPA was first identified for its essential role in DNA replication [38]. However, RPA is multifunctional and also required for modulating DNA repair and recombination [39,40,41,42,43,44,45,46] and maintaining telomeres [47,48,49,50,51,52]. The yeast Rad52 homologous recombination protein antagonizes RPA during homologous recombination. In turn, Rad52 helps to promote Rad51 binding and RPA replacement [53,54,55,56].

RPA participates in checkpoint activation [18,46,57,58,59] and regulates cell cycle progression [60,61,62]. RPA modification patterns are complex and include phosphorylation [57,60,63,64,65,66,67,68,69,70,71,72,73,74,75], acetylation [76], and sumoylation [77,78].

RPA interactions with ssDNA are regulated to minimize an abundance of irreparable and under-replicated substrate, or, to avoid an accumulation of toxic recombination intermediates [44,79,80,81] Due to its important role sequestering and stabilizing ssDNA, RPA binding is a critical DNA damage indicator and sensor [18,82,83]. Not surprisingly, RPA is itself a target of the checkpoint [63,66,67,70]. RPA modification after checkpoint activation may limit a cell’s response to damage [82,84,85].

One of the most common agents used to induce replication stress is hydroxyurea (HU). Hydroxyurea starves the cell for nucleotides and robustly arrests DNA synthesis in wild type cells [86,87]. Hydroxyurea is generally not a lethal challenge, unless the checkpoint response system is disrupted [88]. Other DNA damaging agents such as camptothecin and methylmethane sulfonate generate other forms of stress. Camptothecin (CPT) inhibits topoisomerase activity and generates S-phase specific DNA breaks [89]. Methylmethane sulfonate (MMS) alkylates bases, causing a variety of modifications and adducts that cause DNA replication slowing [90,91].

Mutations in replication proteins may also generate replication stress and cause cancer in human populations and vertebrate models (e.g., [82,92,93]). These genetic mutations disrupt normal replisome function. The single cell fission yeast, *Schizosaccharomyces pombe*, is a convenient model organism to study replication stress. *S. pombe* also has heterochromatic and chromosome features that make it an excellent model for metazoan chromosome instability, e.g., complex centromeres, Thermo-sensitive alleles of essential proteins, such as MCM helicase subunits, cause distinctive forms of stress [35,94]. The majority of temperature-sensitive MCM-helicase mutants (*mcm-ts*) replicate most of their DNA before entering a lethal cell cycle arrest due to accumulated DNA damage, presumably by broken replication forks. In contrast, a *mcm4-degron* mutant has an early replication-failure effect, replicates a small amount of its genome but fails to arrest. These under-replicated *mcm4-degron* cells continue to divide despite accumulating RPA. Stalled and restarted forks are vulnerable to rearrangements (e.g., [95,96,97]) indicating that the effects of stress are intrinsically destabilizing.

In fission yeast, replication stress can be monitored in live cells by imaging foci formed by fluorescently-tagged proteins, most commonly RPA and Rad52 [35,87,98,99,100,101,102,103]. Rad52 is a well-established marker for DNA damage and repair via homologous recombination [54,103,104,105]. While Rad52 foci frequently denote recombination, a subset of Rad52 foci localize to stalled replication forks. These stalled forks lack Rad51 and are presumably not associated with recombination [98,106]. Rad52 signals typically overlap with RPA signal [35,87,98,99,106]. Visual RPA signals are correlated with molecular evidence for ssDNA accumulation and histone H2A.x phosphorylation [19,107,108].

A wild-type population of asynchronously growing fission yeast shows 10%–20% of cells with RPA and/or Rad52 foci [87,103,108]. These are usually single, faint foci that form and resolve during S phase. Few RPA or Rad52 foci accumulate in hydroxyurea-treated wild-type cells during drug treatment. However, wild type cells released from hydroxyurea show a transient increase of RPA and Rad52 signals thirty minutes after release, as the cells complete S phase [87,103]. These symptoms of hydroxyurea recovery are consistent with HR-mediated fork restart or short-track end resection (e.g., [95,103,108,109,110,111,112,113,114]). Longer end resection only occurs on collapsed forks in checkpoint mutants or after prolonged incubation [110]. A second spike of RPA and Rad52 foci are observed 3 h after release, and are likely correlated with replication during the next cell cycle.

In contrast, replication checkpoint mutants including *cds1∆* and *mrc1*∆ steadily increase the numbers of RPA foci during hydroxyurea exposure. The RPA signal observed is higher than the level of Rad52. While Rad52 signal decreases in these mutants after hydroxyurea block and release, RPA levels become more intense over time, generating an bright, pan-nuclear signal [87]. This is consistent with the S-phase checkpoint limiting fork reversal through the activity of Cds1 (CHK1 in humans, Rad53 in *S. cereviseae*) and Mrc1 (CLASPIN homologue) (e.g., [115,116,117]). Replication mutants in the MCM helicase (e.g., *mcm4-ts*, *mcm4-dg*) show steadily-increasing accumulation of RPA and Rad52 during replication stress. These foci do not resolve after replication stress ends [35,87].

**Figure 2:**
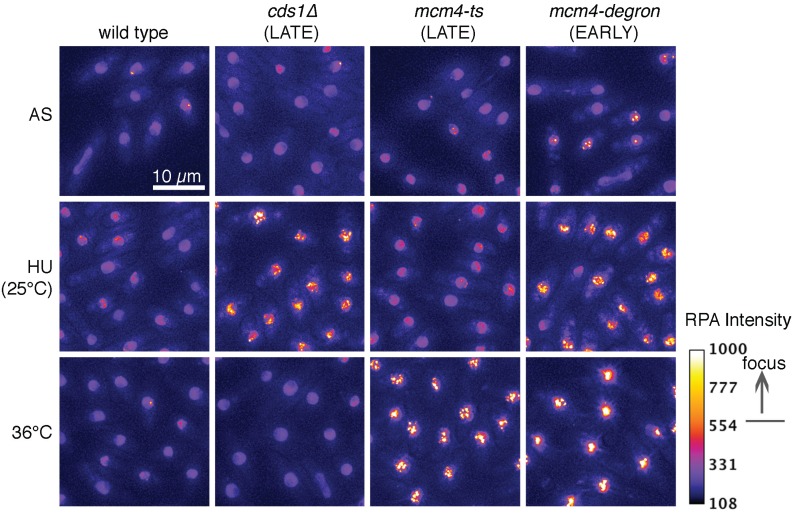
RPA intensity and localization patterns depend on stimulus and effect, and are visualized in live cells with RPA-CFP. For example, *cds1∆* cells exposed to hydroxyurea (HU) for 3 h at 25 °C begin to accumulate pan-nuclear RPA signal, but do not form RPA foci when shifted to 36 °C for 4 h. In contrast, *mcm4-ts* cells only accumulate RPA foci after 4 h at 36 °C, but not in HU. Both of these mutants exhibit a late replication arrest, and develop widespread nuclear RPA signal, although the *mcm4-ts* mutant retains a punctate pattern while RPA coalesces into a pan-nuclear bolus in *cds1∆* + HU. In contrast, the *mcm4-degron* is an early-replication arrest phenotype after 4 h at 36 °C, forming discrete and bright RPA foci that we believe are a signal of clustered early origins arrested in S-phase. The wild-type control cells fail to accumulate RPA signal in either HU or temperature conditions. We use a heat map to depict RPA, and foci above threshold are orange to yellow signal (heat map scale, right). Cells in these pictures were grown in minimal medium, and incubated in 12 mM HU at 25 °C, or in a 36 °C water bath.

Significantly, the patterns of RPA accumulation are distinct for different forms of replication stress, ranging from multiple small foci in *mcm4-*ts, to a single large focus in *mcm4-dg*, to massive pan-nuclear staining in *cds1∆ + HU* [35,87] (Figure 2). This indicates that our concepts of “replication stress” and “fork collapse” likely encompass a range of different molecular structures, depending upon the challenge. There are different patterns of division after challenge as well. The *mcm4-M68* mutant replicates much of its DNA during temperature shift and following release, and then enters a damage-checkpoint dependent cell cycle arrest. In contrast, the *mcm4-ts-degron* mutant synthesizes little DNA, and evades the checkpoint. The *mcm4-degron* cells continue to divide, causing DNA mis-segregation, aneuploidy and formation of apparent micronuclei. A subset of *cds1∆ + HU* cells also continues division following release, although the majority of cells remain arrested. Environmental conditions play a role and alter RPA accumulation and/or stability. In our work we have seen that replication instability induced at the same time as incubation at high temperature (37 °C) may alter RPA focus distribution and stability. This effect of temperature on RPA distribution may indicate potential changes to the DNA damage checkpoint, such as those reported by [118].

Finally, we examined *rad51∆* to compare replication-checkpoint deficient cells with a repair-deficient strain*.* We observed substantially higher endogenous levels of RPA and Rad52 in asynchronously growing *rad51∆* cells, indicating a baseline of replication stress. The number of RPA and Rad52 foci increased after HU treatment. In contrast to the large RPA masses that accumulated in the checkpoint mutants, RPA remained in discrete puncta in the *rad51∆* strain. RecA, the bacterial Rad51 homologue, interacts with the bacterial RPA homologue, single strand binding protein (SSB) [119] to regulate the transfer of ssDNA to RecA. This suggests that even as the *rad51*∆ strain forms DNA damage in HU, RPA accumulation is blocked, as cells cannot transfer ssDNA to recombination repair.

Clearly, the replicative MCM-helicase is important in generating ssDNA. Yet, conditions that eliminate MCM function can also generate RPA foci [35,87]. This may reflect activity of other helicases or nucleases, end resection, or strand invasion during repair and fork restart.

## 4. Association between ssDNA and DNA Damage

Although a threshold level of ssDNA exists that determines DNA damage checkpoint activation, the amount of ssDNA required to reach the threshold is not clear. Evidence suggests that RPA is limiting for the checkpoint response, while excessive ssDNA may exhaust RPA protective capacity [85]. Intriguing data from *S. cerevisiae* indicate that the enigmatic Rif1 protein may antagonize RPA early in the response to stress [81].

Excess ssDNA is associated with double strand breaks following replication stress, which may be linked to the cells entering mitosis in the presence of ssDNA [30,120,121,122,123]. There are dramatic consequences to undergoing nuclear division with persistent DNA damage or ongoing replication: prolonged M phase, lagging chromosomes, chromosome bridges, and cohesion fatigue [124,125,126]. It is likely that the contributions of ssDNA are under-recognized in many situations, since ssDNA can reduce the signal associated with dsDNA. Most dsDNA dyes are intercalating agents (e.g., [87]). Recent studies identified ultrafine anaphase bridges (UFB), which cannot be seen with typical DNA dyes or histone labels, but are visualized by binding by proteins including RPA and the BLM helicase [3,8,29,35]. This suggests that UFBs are threads of ssDNA. Some evidence suggests that UFBs result from under-replicated DNA at fragile sites (e.g., [8,9]). Clearly these RPA-coated structures evade typical checkpoint mechanisms, allowing mitosis to occur. This may reflect ssDNA formed as a result of mitosis (and, thus, RPA binding after mitotic entry, past the point of checkpoint restraint), or that not all forms or structures of RPA are capable of nucleating checkpoint activation.

**Figure 3:**
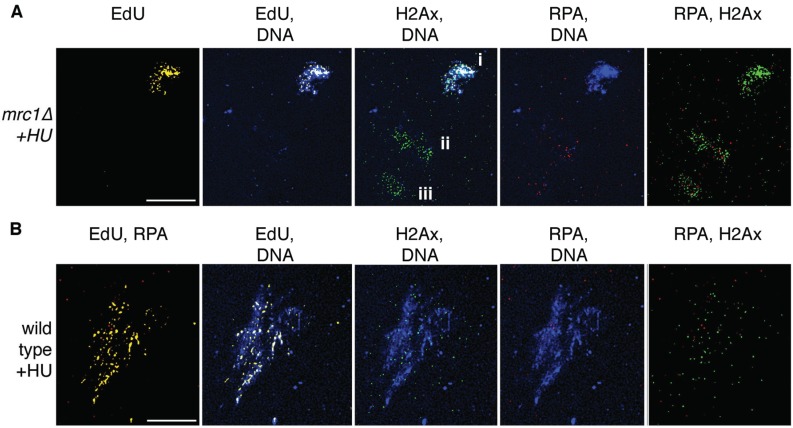
Detection of samples is dependent on the channel used to find the spread nuclei. (**A**) checkpoint-deficient mrc1∆ cells were exposed to HU and EdU to determine synthesis during HU block. After immunofluorescent detection, samples were located using the DNA synthesis marker (EdU, yellow). In this field, three spread nuclei are present, but only one is EdU-labeled (i). The others (ii, iii) are highly decorated with DNA damage signal (H2Ax, green), and ssDNA (RPA, red), but have little DNA signal (DAPI, blue). In this case, only the highly-replicated sample would have been found if the others were not nearby. Scale, 15 µm; (**B**) as a comparison, wild-type cells were treated with HU and EdU as in A, and processed identically. In this case there is sporadic EdU label, and very little RPA indicating that forks are restrained and/or arrested. There is high DAPI signal indicating that dsDNA is stable, and the sprinkling of DNA damage (H2Ax) suggests that the stalled and arrested forks have activated the replication checkpoint and are being monitored. Scale 10 µm.

There are practical and technical considerations in determining how ssDNA formation, RPA binding, and the DNA damage response are linked. For example, how does one choose the signal to detect when hunting for samples in a sparse field? An example of this is in spread nuclei, prepared and stained to detect dsDNA, H2Ax, RPA, and synthesis using a nucleoside analogue. Nuclei that have not spread sufficiently tend to retain the signal for the first three markers that is likely non-specific. Thus, it is vital to detect samples of similar quality and resolution. However, what channel should be used to find spread nuclei? Using dsDNA signal will exclude low-staining samples that may contain high amounts of ssDNA and/or H2Ax; in this case, the highly damaged DNA decreases the dsDNA signal using DAPI stain (Figure 3A). Alternatively, looking only for replicated samples may exclude those that fail to synthesize large amounts of DNA (Figure 3), and so on. The important issue is to compare like sample to like, and to examine all channels under study in order to detect samples.

DNA replication fork collapse is a more dynamic and longer process than previously anticipated [87]. Ongoing synthesis in checkpoint mutants released from drug treatment suggests that replication forks continue progression before finally losing their ability to synthesize DNA, at a time we call the “Fork Collapse Point”. This is an execution point that describes a point of no return, when forks are no longer able to function and/or synthesize DNA. The burden of ssDNA that accumulates during HU treatment of checkpoint mutants, and the fact that cells require RPA and Rad52 to survive replication stress, illustrates that bulk genome replication is not the only source of replicative stress during HU treatment, and this may account for the different patterns of RPA we observe. The full consequences of ssDNA accumulation may not develop until after replication stress, as cells attempt to recover and re-enter the cell cycle. It may be that division in the presence of unresolved ssDNA is the primary cause of genome instability (e.g., [120,123]).

## 5. Conclusions

We are poised to explore how RPA integrates its roles as a sensor, a signal, and a repair mediator after replication stress using a fission yeast model organism. By signaling damage and then mediating a transition to recombinational repair, ssDNA, and RPA likely play a pivotal role in preventing large-scale genomic mutations that contribute to cancer establishment and progression. Substantial progress in the last few years has led to exciting new questions. How is ssDNA protected to minimize the risk of DNA damage and rearrangement during stress? How does the presence of ssDNA impact the fidelity of chromosome segregation during mitosis? How does this impact genome stability in later cell cycles? Given the substantial conservation of mechanisms that respond to replication stress, studies of ssDNA and RPA are likely to provide fundamental insights into many human diseases associated with genome instability.
